# Determination of the Stifle Angle at Standing Position in Dogs

**DOI:** 10.3390/vetsci9110644

**Published:** 2022-11-21

**Authors:** Thomas Giansetto, Pierre P. Picavet, Michaël Lefebvre, Marc Balligand

**Affiliations:** Department of Companion Animal Clinical Sciences, Faculty of Veterinary Medicine, University of Liège, 4000 Liège, Belgium

**Keywords:** stifle, cranial cruciate ligament rupture, kinetics, kinematics, X-rays

## Abstract

**Simple Summary:**

The cranial cruciate ligament rupture is one of the most common orthopaedic diseases encountered in dogs. Surgical techniques have been developed to stabilize the stifle, with an overall accepted benefit of tibial osteotomies, among which is the tibial tuberosity advancement. Prior to surgery, the required tibial tuberosity advancement must be determined on a strict lateral radiographical view of the affected stifle with femur and tibia at an angle of 135° as initially recommended. We sought to determine if this particular stifle angle around mid-stance phase was similar among multiple dog breeds. A mean value of stifle angle of 145° was obtained. Mean stifle angle at mid-stance phase in a healthy dog is regularly higher than 135° and is likely breed and individual dependent. The pre-operative measurement of the required advancement made on stifles in 145° extension, a value close to full physiological extension, could contribute to decreasing the incidence of late post-operative meniscal lesion, consecutive to the underestimation of the tibial tuberosity advancement.

**Abstract:**

Background: The cranial cruciate ligament rupture is one of the most common orthopaedic diseases encountered in dogs. Surgical techniques have been developed to stabilize the stifle, with an overall accepted benefit of tibial osteotomies among which is the tibial tuberosity advancement (TTA). Prior to surgery, the required TTA must be determined on a strict lateral radiographical view of the affected stifle with femur and tibia at an angle of 135° as initially recommended. This value, initially determined in only two dog breeds, has been considered the mean standard value of the canine stifle angle during the mid-stance phase. Methods: We sought to determine if this particular stifle angle around mid-stance phase was similar among multiple dog breeds. We built up a custom-made radiographic system for stifle angle measurement in standing dogs. Results: A mean value of stifle angle of 145° was obtained. Mean stifle angle at mid-stance phase in a healthy dog is regularly higher than 135° and is likely breed and individual dependent. Conclusions: The pre-operative measurement of the required TTA made on stifles in 145° extension, a value close to full physiological extension could contribute to decreasing the incidence of late post-operative meniscal lesion, consecutive to underestimation of TTA.

## 1. Introduction

Cranial cruciate ligament rupture (CCLR) is one of the most common orthopaedic diseases affecting the pelvic limbs in dogs [1,2]. While the definitive causes are still under debate, it is accepted that this affliction is predominately degenerative in dogs [3]. Forces exerted on the stifle and more precisely on the cranial cruciate ligament (CCL) vary throughout the stance phase. Based on the theory of Tepic, the resulting force applied on the tibia during weight bearing has a direction close to the direction of the patellar tendon [4]. The physiological cranial tibial thrust increases with the stifle joint forces, reaching a maximum at mid-stance phase [3]. The stifle angle value leading to high CCL load has been reported in the literature to be the one close to mid-stance phase (beginning of the propulsive phase) with a mean value of 135° measured in dogs of only two breeds, crosses of Labrador and German Shepherd [5].

Several treatments for incompetent CCL have been extensively investigated with tibial osteotomy techniques showing the best long term results [6,7]. TTA, based on the theory by Tepic, is one of them [3,4]. To determine the required TTA, the tibial plateau slope and its angle with the femoro-patellar tendon should be preoperatively determined on strict mediolateral stifle radiographic projections [8].

Persistent stifle instability post-TTA has been reported as a cause for postliminary meniscal tears following preoperative measurements made on stifles flexed at 135° [9]. Our hypothesis is that 135° might be an underestimation of the mid-stance phase angle in certain individuals, possibly contributing to insufficient TTA during surgery.

The purpose of this study was to measure the stifle angle on standing position in various dog breeds using a set of radiographs. Although previous studies have used reflective markers attached to the skin to determine limb axes and joint angulations, these markers introduce bias simply because the skin can move independently from bones leading to significant variations in angle measurement [10,11,12].

## 2. Materials and Methods

Owned dogs were recruited for the study after owner consent. Breed, age, weight, size and sex of each animal were collected. Inclusion criteria included absence of any orthopaedic or neurologic anomaly. Orthopaedic and neurological examinations were performed on all dogs by one of the authors (M.L.).

### 2.1. Materials

**Figure 1:**
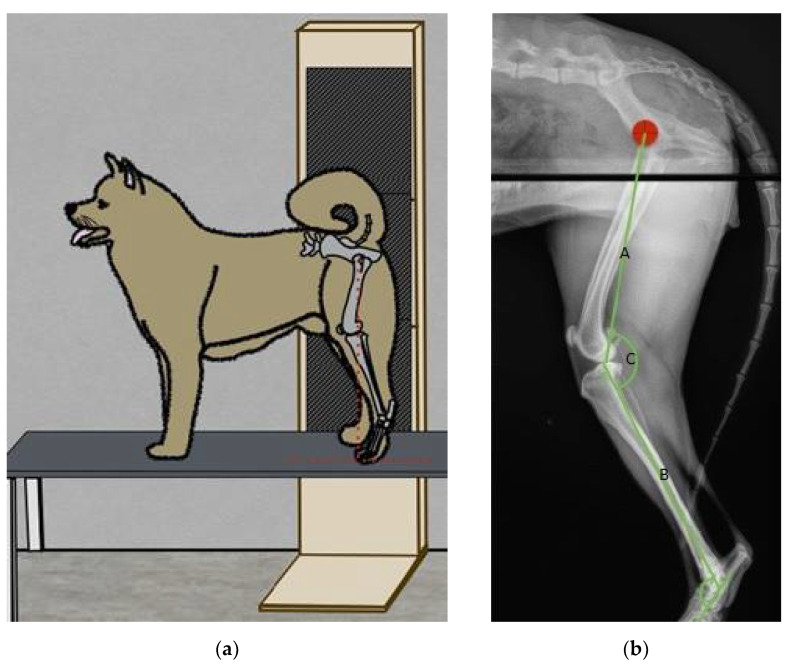
(**a**) Schematic representation of the custom-made cassette holder and positioning of the dog during radiograph processing. (**b**) Mechanical axis on radiographs (A: femur; B: tibia; C: stifle angle).

For each dog, multiple radiographic mediolateral projections (between 1 and 4) of the entire pelvic limbs were taken while the dog was standing at rest. A wooden custom-made vertical cassette holder (162 × 61 cm) was used. This holder enabled us to vertically stack three Cr Agfa cassettes (46 × 38 cm) with an overlapping of 4.5 cm of each cassette in order to avoid any discontinuity in the final radiographical reconstruction. A portable device (Portable X-ray machine HF 80/15) attached to a custom-made metal base was used to take radiographs. The beam was centered on the stifle (Figure 1a). The distance between the X-ray device and the cassette holder was 225 cm. Using image processing software (Photoshop ND), one image containing the entire pelvic limb of each dog was obtained.

### 2.2. Measures

Mechanical bone axes were used in order to determine joint angulation. On lateral views, the femoral mechanical axis was defined by the line between the centre of the femoral head and the centre of the femorotibial joint. The tibial mechanical axis was defined by a line joining the tibial intercondylar eminences to the talus center. The tarsal mechanical axis was defined as a line running through the talus center parallel to the longitudinal axis of the metatarsal bones (Figure 1b).

The foot ground contact point was directly below the hip and pelvic limbs were superimposed.

### 2.3. Statistical Analysis

A Spearman correlation test was used to find a correlation between the femorotibial and tibiotarsal angles and a linear regression was used to evaluate the effect of weight on angles.

## 3. Results

Twenty-four dogs were included in the study. Breeds included mixed breed (n = 8), Weimaraner (n = 1), Mastiff (n = 1), Dutch Canardier (n = 1), German Shepherd dog (n = 2), White Swiss Shepherd Dog (n = 1), Belgian Shepherd (n = 3), Siberian Husky (n = 1), French bulldog (n = 2), Bouvier des Flandres (n = 1), Jack Russel Terrier (n = 2) and Border Collie (n = 1). After reviewing the radiographs, three cases were excluded from the study because of mispositioning. The femorotibial angles were between 129.5° and 156.6° (145.3°+/−7.9). Tibiotarsal angles were between 122.5° and 147.7° (134°+/−9.1) (Table 1).

## 4. Discussion

The main goal of this study was to determine the stifle angle at the mid-stance phase of dogs in order to allow better pre-operative planning in case of CCLR treated by TTA. The results provided evidence that the stifle angle at the mid-stance phase varies in dogs, with a mean value of 145°.

Previous studies already showed this inter-individual difference but were using retroreflective markers, leading to the introduction of a different bias as previously described [11]. Indeed, these markers are usually circular in shape and studies do not explain which part of it is used for the reference point. Furthermore, a study showed that the location of the markers on the skin itself could differ from 0.4 to 1.2 cm among repeated cycles of measurement. Such a displacement can cause significant differences in angle measurements [10], particularly in small individuals. Radiographs instead lead to more accurate measurements with clear bony reference points. It is of primary importance for the X-ray beam to be perpendicular to the limb to allow meaningful angle measurements.

A previous study reported the stifle angle during stance phase at rest in two groups of dogs, all with CCLR [13]. We chose to use healthy dogs instead, in order to better mimic the physiological situation. It is known that with CCLR or painful stifle, the limb is maintained slightly flexed in order to relieve pain [14,15]. In the previous study, the mean stifle angle was 138° against 145° in our study.

During gait, ground reaction forces increase, with speed [13] having an impact on joint angles in comparison with the standing position. It could therefore be more relevant to measure the stifle angle during the gait. Loading the back of dogs before taking radiographs at rest could have mimicked this larger force exerted on the stifle. Unfortunately, our dogs could not stand such load. Nevertheless, a study recorded the stifle angle at different gaits and showed no significant difference in stifle angle during the various gaits (walk and trot) [16].

## 5. Conclusions

In conclusion, our results indicate that the stifle angle at rest in a position corresponding to the mid-stance phase is regularly higher than the 135° previously published. It is close to 145° and is individual dependent. As the calculation of the required TTA on a 135° stifle angle leads to underestimation of TTA, we recommend taking the pre-operative radiographs with the stifle in full extension.

## Figures and Tables

**Table 1:** Breed, weight, femorotibial angles, tibiotarsal angles and tibial plateau angles.

Animal	Breed	Weight (kg)	Mean Femorotibial Angle (°)	Mean Tibiotarsal Angle (°)	TPA Angle (°)
1	Dutch canardier	11	132.9	136.9	21.4
2	Mixed breed	27.9	133.6	128.6	24.4
3	Berger Blanc Suisse	41.5	136.9	125.4	18.8
4	Mixed Breed	18	142.4	128.6	19.5
5	Siberian Husky	20.9	142.5	113.8	15.4
6	Belgian Shepherd	28	143	131.9	17.8
7	Mixed breed	8.5	144.3	127.5	23.9
8	Mixed breed	20.4	145.3	121.4	35.7
9	Belgian Shepherd	31.7	146.7	134.7	14.3
10	Weimaraner	37.5	147.3	137	33.9
11	Belgian Shepherd	22	150	140.5	30.2
12	Border Collie	24.5	150.8	147.7	18.7
13	Mastiff	62	151.8	145.8	19.1
14	Bouvier des Flandres	31.2	155.6	135.2	24.1
15	French Bulldog	7	156.6	127.2	28.9
Mean value		26.1	145.3	132.1	23.1

No significant correlation between the femorotibial and tibiotarsal joint angles was noted (r = 0.38 and *p* = 0.15). No significant effect of weight on femorotibial, tibial plateau and tibiotarsal angles was detected by linear regression (*p* = 0.68, *p* = 0.46 and *p* = 0.14, respectively).

## Data Availability

The data presented in this study are available in: Determination of the stifle angle at standing position in dogs.
